# Microinvasive mitral valve repair with transapical mitral neochordae implantation

**DOI:** 10.3389/fcvm.2023.1166892

**Published:** 2023-07-28

**Authors:** Emma Bergonzoni, Augusto D’Onofrio, Florinda Mastro, Gino Gerosa

**Affiliations:** Cardiac Surgery Unit, Department of Cardiac, Thoracic, Vascular Sciences and Public Health, University of Padua, Padua, Italy

**Keywords:** microinvasive cardiac surgery, mitral valve repair, transapical mitral valve repair, transapical neochord implantation, transcatheter neochord implantation

## Abstract

Microinvasive cardiac surgery includes procedures performed off-pump, on the beating heart, with limited or absent skin incision, and those that rely on live imaging techniques. Transapical off-pump beating heart neochordae implantation allows the repair of severe mitral valve regurgitation due to leaflet prolapse or flail with live three-dimensional echo guidance. This procedure has shown good results for up to 5 years and can be considered as a valid alternative to conventional surgery in selected patients with high prediction of success based on clinical and anatomical considerations. The aim of this review is to describe the devices, indications, patient screening process, clinical and echocardiographic results, and future perspectives of this procedure.

## 1. Introduction

Microinvasive cardiac surgery includes procedures performed off-pump, on the beating heart, with limited or absent skin incision, and those that rely on imaging techniques. The difference between minimally invasive and microinvasive cardiac surgery is that the former always needs cardiopulmonary bypass, cardioplegic arrest, and general anesthesia while the latter can be carried out off-pump, on the beating heart, and with local anesthesia only. Examples of microinvasive cardiac surgery are transcatheter aortic valve implantation (TAVI) and edge-to-edge transcatheter mitral valve repair. These procedures mimic conventional cardiac surgery operations without the need for extracorporeal circulation, cardioplegic arrest, and opening of cardiac cavities and can be carried out with a microinvasive approach. Transapical microinvasive mitral valve repair with neochord implantation (NC) was introduced into clinical practice in 2012. This procedure consists in correcting mitral leaflet prolapse by positioning, through transapical access, one or more artificial chordae, and by pulling them under live 3D TEE guidance. There used to be two commercial devices: NeoChord DS1000 (NeoChord Inc, St. Louis Park, MN, USA) and Harpoon (Edwards Lifesciences, Irvine, CA, USA). Since the latter has been recently withdrawn from the market and is no longer available for clinical use, in this article, we will mainly focus on the NeoChord device.

This article will discuss the indications and contraindications for NC, patient selection, technical aspects, learning curve, clinical and echocardiographic outcomes, and future perspectives.

### 1.1. Indications and contraindications

Degenerative mitral valve regurgitation is the most common heart valve disease seen in adults. It affects more than four million people in Europe and the United States and it has an incidence of 1%–2% of the Western population (1). According to ESC guidelines, severe primary mitral regurgitation *must be* treated surgically when the results are durable, when the patient is symptomatic, operable, and not high risk, and when the patient has no symptoms but the left ventricle is dysfunctional (LVESD > 40 mm and/or LVEF < 60%). Otherwise, surgery *should be* considered in asymptomatic patients with normal left ventricular function and atrial fibrillation secondary to mitral regurgitation or pulmonary hypertension (PAPs at rest >50 mmHg) or in low-risk asymptomatic patients with normal ventricular function and significant left atrial dilatation (volume index >60 ml/m^2^ or diameter >55 mm) (2). The gold standard surgical option for chronic primary mitral regurgitation is repair over valve replacement because mitral valve repair showed big advantages in terms of preservation of postoperative left ventricular function, perioperative mortality, avoidance of anticoagulation, and long-term survival (3). Currently, there are several surgical techniques among which the surgeon can choose depending on the type of lesion causing the valve disease. In order to simplify the anatomical and functional evaluation and the surgical possibilities of a regurgitating mitral valve, a “functional classification” was introduced by Carpentier, and it was based on the analysis of the motion of the leaflets by echocardiography and direct vision of the surgeon. This classification is divided into three types depending on whether the motion of the leaflets is normal (type I), increased (type II), or decreased (type III). Each type of defect has different causes and can involve different components of the MV apparatus (i.e., type II could be determined by an elongation or rupture of chordae or papillary muscle) and has well-defined surgical possibilities in order to be repaired (4). According to these basic but paramount concepts, the repair of mitral valve regurgitation using transapical microinvasive neochordae implantation is indicated in patients with severe chronic degenerative mitral valve regurgitation and Carpentier's type II lesion, in particular chordal elongation and/or rupture causing leaflet prolapse/flail, in order to *restore* the competence of the valve (5). Together with severe MVR and leaflet prolapse/flail, correct patient selection requires a careful assessment of other aspects such as annular diameter and left ventricular volume.

### 1.2. Patient selection for NC

Since the introduction of this new technology, patients can be divided into four groups according to regurgitating MV anatomy: “type A” with isolated central posterior leaflet flail/prolapse, “type B” with posterior multi-segmental flail/prolapse, “type C” with anterior or bi-leaflet prolapse/flail, and “type D” with paracommisural flail/prolapse or any significant leaflet or annular disease such as calcification (6, 7). This classification is very important because it is strictly connected to early and late results. Colli et al. reported significant differences in residual mitral regurgitation between the four groups with better results in type A and B patients (92% in type A, 89% in type B, 80% in type C, and 80% in type D) (6). Similar results were reported also by other studies both at 6-month and 1-year follow-ups (8, 9).

**Figure 1:**
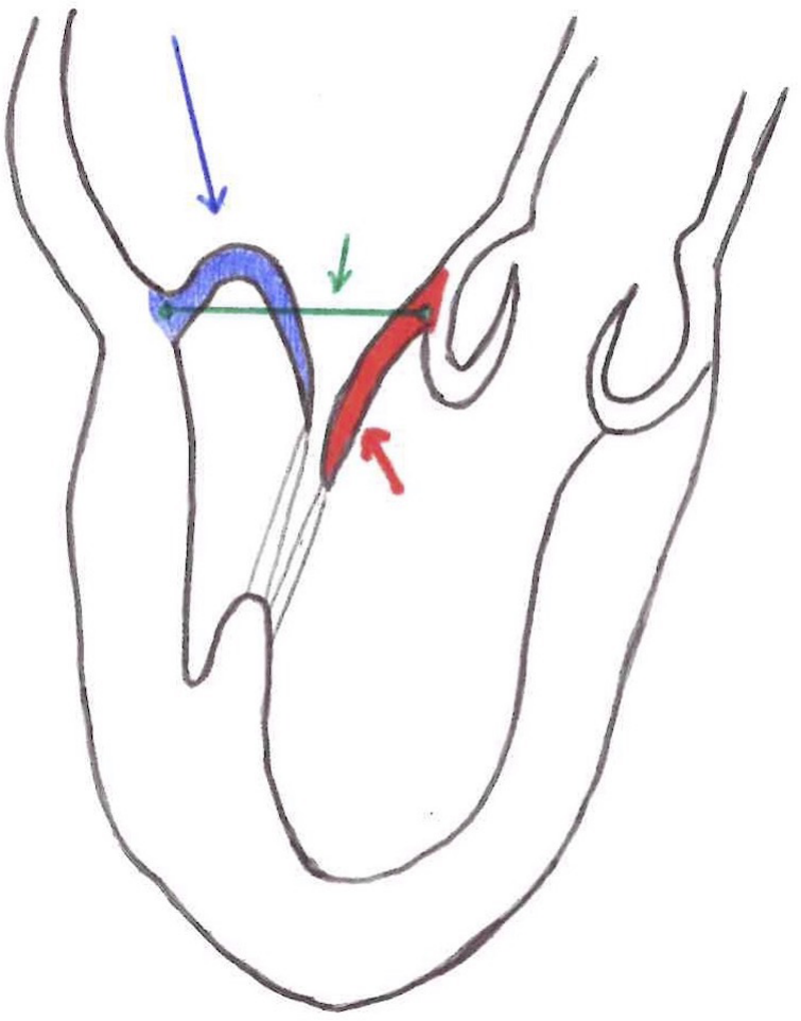
Red line = anterior mitral leaflet length. Blue line = posterior mitral leaflet length. Green line = anterior-posterior diameter. LAI (leaflet-to-annulus index) = (posterior mitral leaflet length + anterior mitral leaflet length)/ anterior-posterior diameter.

Another important parameter for patient selection is the leaflet-to-annulus index (LAI). LAI is calculated as the ratio between the sum of the height of the anterior and posterior leaflet and the anteroposterior diameter and represents the amount of excess leaflet tissue that can potentially be used to recreate and correct leaflet coaptation (Figure 1). The cut-off value is 1.2, which corresponds to a 20% excess of leaflet tissue, and it is associated with mitral regurgitation ≤mild at 1-year follow-up (6, 10). Annular diameter is inversely related to the feasibility of the procedure: a large annulus determines low LAI. Generally, in the early severe MR phase, the annulus is not enlarged or flat. Thus, it is crucial that NC, which does not address the mitral annulus, is performed before annular enlargement occurs. However, as outlined below, positive annular remodeling has been observed after the procedure but there is not enough data to draw final conclusions. Annular enlargement should not be considered an absolute contraindication to NC *per se* because, if leaflet tissue is abundant, LAI will still be in the feasibility range.

**Figure 2:**
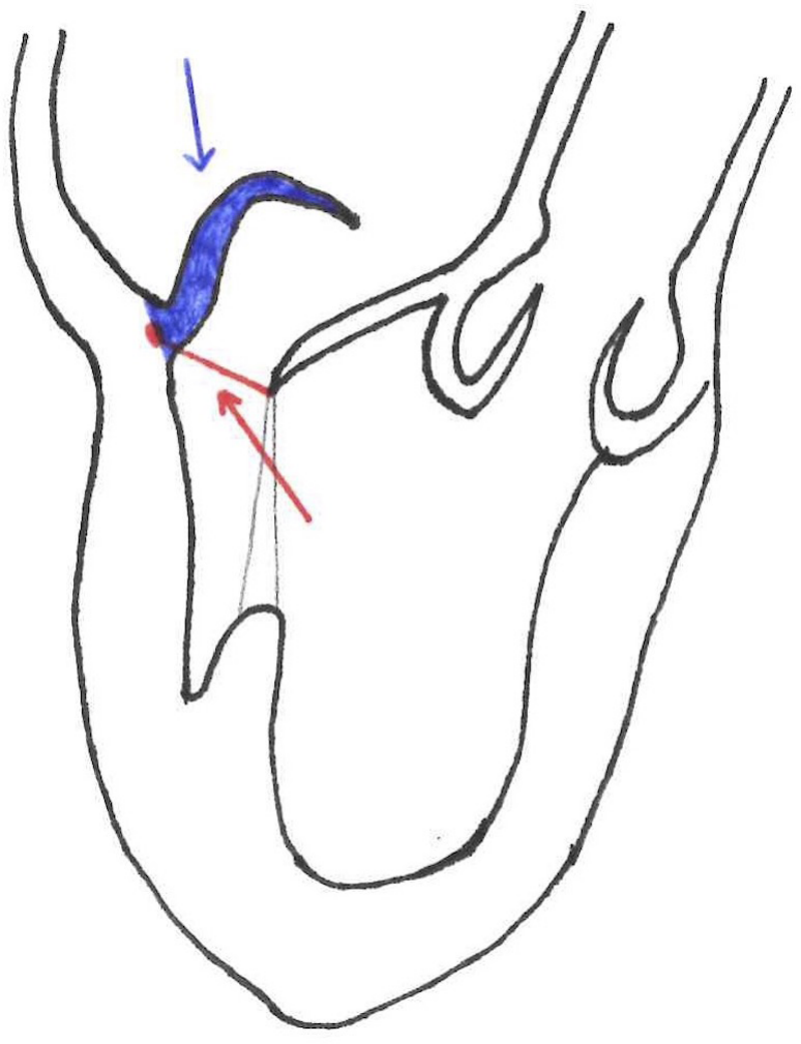
Blue line = posterior leaflet length. Red line = gap size. Gap Ratio = posterior leaflet length/ gap size.

Another inclusion parameter focused on the tissue is the gap ratio which is defined as the ratio of the height/length of the prolapsing segment of the posterior leaflet to the gap between the coaptation surface of the anterior leaflet and the hinge point of the base of the posterior leaflet, measured at the peak systole. The cut-off is a gap ratio of 1.5:1 or more in order to have adequate posterior leaflet tissue to allow sufficient coaptation and effective abrogation of mitral regurgitation after repair (Figure 2) (11).

In a study published in 2019, Colli et al. analyzed the possible mechanisms of recurrent regurgitation after neochord implantation. In total, 52 patients with recurrent moderate or severe MR were considered as Not Expected Surgical-Like Results (NESLR)-Redo and NESLR-MR2 (respectively reintervention or moderate MR 2+). They also divided the patients by onset of the regurgitation and by jet orientation and direction. After that, the authors analyzed the specific mechanisms of NESLR. Among the key points responsible for recurrent regurgitation, patient selection (17.3%), technical issues (28.8%), progression of baseline disease (15.4%), left ventricle reverse remodeling (1.9%), excessive over-tensioning (35.8%), and PML curling (30.8%) were identified. Detailed evaluation of valve anatomy (in particular, annular calcification which is associated with more rigid and fragile leaflets) and a badly calculated LAI were the main problems occurring during patient selection. Furthermore, in this and in other studies, cases of neochord rupture were reported. Reasons can be mainly related to prolonged friction between different materials (ePTFE, polypropylene, pledgets), excessive over-tensioning, and inadvertent neochordae damage during intraoperative manipulation but no clear data to assess this issue is available (12, 13).

Exclusion criteria for NC implantation through a transapical approach are previous left chest surgery, pleural disease (7), active endocarditis, secondary mitral regurgitation, unfavorable anatomy (i.e., heavily calcified valves, significant leaflet tethering, presence of multiple or complex color Doppler jets, significant tricuspid or aortic valvular disease, leaflet perforation, and less than 5 mm flail overlap of the diseased leaflet with respect to the normal one) (3), and severe left ventricular dysfunction (14). Table 1 summarizes the preoperative factors that need to be evaluated during the screening process and that may predict ideal, good, possible, or unsuitable candidates for NC.

**Table 1:** Preoperative factors that need to be evaluated during patient screening.

	Ideal	Good	Possible	Unsuitable
Disease location	Isolated central posterior leaflet P2	Wide posterior multisegment P1-P2 or P2-P3	Anterior or bileaflet A2 or 2-P2	Commissural/paracommissural
Leaflet tissue disease	Important leaflet redundancy and thickness	Moderate leaflet redundancy and thickness/forme fruste	No leaflet redundancy, thinned leaflet	Rigid leaflet, reduced mobility, restrictive/rheumatic
LAI (leaflet-to-annulus index)	>1.2	1.15–1.19	1.1–1.4 (more anterior ventricle access site is suggested)	<1.1
Flail width	10–20 mm	20–30 mm	>30 mm	<8 mm
Calcification	NO	NO	Mild annular	Heavy/exstensive annular and leaflet
Indexed left ventricle end-systolic volume (iLVESV)	12–30 ml/m^2^	31–40 ml/m^2^	40–55 L/m^2^	>55 ml/m^2^

The biggest concern related to NC is the lack of annular stabilization which is a well-established predictor of repair durability in conventional surgery. However, many echocardiographic analyses showed, during NC follow-up, positive modifications of the valve and of the left ventricular geometry, and in our experience with trans-apical NC, failure has never been related to annular dilatation (13, 15). This is still a matter of debate and further data are needed to definitively assess the impact of no annular stabilization during NC.

### 1.3. NeoChord DS1000

**Figure 3:**
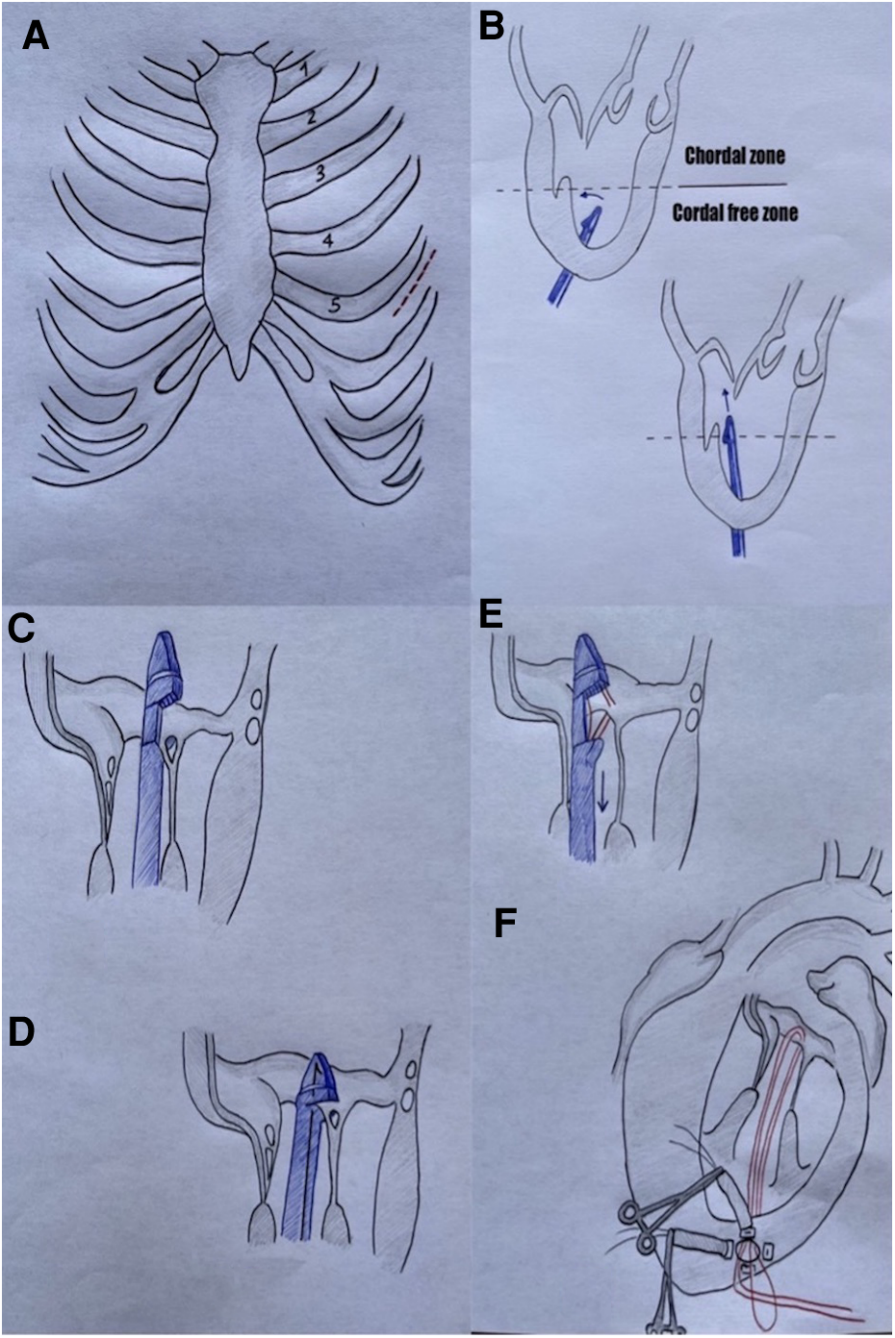
**A** = mini-thoracotomy site; **B** = NeoChord system navigates in the LV towards mitral valve; **C** + **D** = NeoChord grasps posterior leaflet; **E** = the needle is pushed through the tissue; **F** = a girth hitch knot is performed outside the LV.

The device consists of a single-use, hand-held device designed to load and deploy ePTFE sutures through exchangeable cartridges and a tethered leaflet verification display that confirms leaflet capture in the distal clamp of the instrument through four fiber optic lights that turn from red to white. During the operation, a stand-by cardiopulmonary bypass machine is recommended. The patient is under general anesthesia, mechanically ventilated through a double-lumen endotracheal tube, and rolled onto their right side in order to better expose the LV apex. The surgeon performs a left anterior mini-thoracotomy in the fifth intercostal space. After the left lung is excluded, the correct LV access site is identified by pushing one's finger 2–3 cm laterally and posteriorly from the true apex under 2D-TEE imaging. The correct identification of the entry site allows one to face the MV directly with the delivery system and to navigate through the subvalvular apparatus safely. Two concentric purse-string sutures of 2–0 MH polypropylene are placed in a crown-like manner with 4–5 large custom-made Teflon pledgets. Recently, we moved towards a more simple set-up with two perpendicular apical mattress sutures. Heparin is then given at a dose of 100 UI/Kg to reach an ACT of >300 s In the meantime, the instrument is loaded with one ePTFE suture and its specific needle. The surgeon performs transapical ventriculotomy with an 11 blade scalpel and prepares the entry site with a closed long straight clamp. When the device enters the LV, the purse strings are slightly tightened to reduce blood leakage. The NeoChord DS1000 is directed towards the left atrium with TEE guidance, avoiding the subvalvular apparatus by keeping the instrument under the A2-P2 segment. Once the mitral valve is passed, echocardiography imaging switches from 2D to 3D and the device is moved towards the prolapsing leaflet. When an appropriate position has been reached, the jaws of the device are opened, and the leaflet is grasped; the grasp is successful if all four fiber lights on the optic monitor turn from red to white. At this moment the surgeon keeps a firm hold of the device and carefully pushes the needle through the leaflet. Then the needle is retracted until the suture loop exits the instrument and the device is pulled out from the ventricle with the jaws opened while the two ends of the suture are gripped manually. The surgeon applies tension to the neochord and a girth hitch knot is secured to the leaflet, locking one head of the suture on the valve segment while the two ends remain outside of the LV. Additional chordae can be added by repeating the procedure in order to achieve maximal competence of the mitral valve (generally at least three sutures are needed to distribute the tension, avoiding excessive mechanical stress). When a satisfactory number of chordae are applied, the apical purse string is tied, and an “eye needle” is used to pass through a round rigid Teflon pledget (big enough to cover the ventriculotomy) and all the neochordae. The pledget is then fixed with a 4-0 prolene suture over the ventriculotomy and tourniquets are placed over every neochord. Neochordae tensioning is then performed under TEE guidance in order to achieve good leaflet coaptation (16, 17). Reverse remodeling has been observed and described after the NeoChord procedure. These modifications in LV volume could lead to a relative elongation of the implanted neochordae, defined as pseudoelongation, causing MR recurrence. Therefore, overtensioning of the neochordae is applied intraoperatively until the appearance of a minimum pseudoprolapse of the untreated leaflet is observed and the PML results are more vertical, but valve competence is still maintained (Supplementary Video S1). This expected trace MR usually disappears within 30 postoperative days (12, 13). Once the neochordae are properly tensioned, they are securely tied to the epicardial pledget. The pericardium is partially closed, a single chest tube is inserted, and the mini-thoracotomy is closed (17) (Figure 3).

Posterior access is recommended to achieve more physiologic chordal implantation and well-distributed tension over the chordae and the leaflet edge (12).

### 1.4. Harpoon

**Figure 4:**
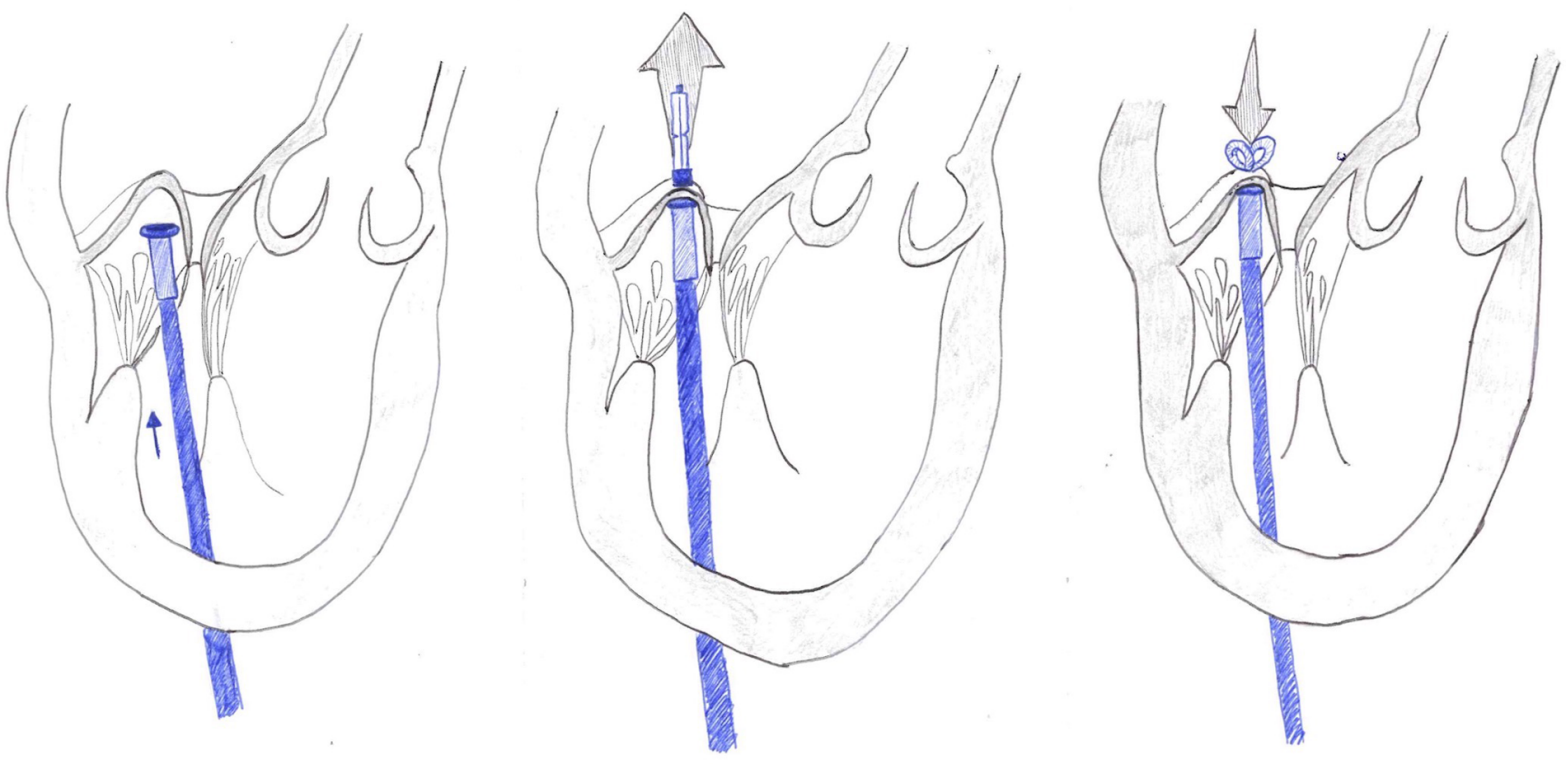
The main surgical steps during neochord implantation with harpoon.

The Harpoon Mitral Valve Repair System (H-MRS) is composed of a 12-Fr introducer, with a hemostatic valve, and the delivery system (a pre-loaded 19-Fr external diameter rigid shafted instrument) is characterized by an atraumatic end effector at the distal end which can stabilize the device on the ventricular side of the mitral valve leaflet at the targeted implantation site. Access is similar to the NC one in the fifth intercostal space. Once the introducer is inserted in the LV apex, the delivery system is directed toward the ventricular side of the prolapsed leaflet segment. The device is then steered under the leaflet target point and the leaflet is stabilized on the tip of the instrument, the needle is engaged, and the knot (double-helix knot) is deployed (targeting goals include placement of the ePTFE knots close to the free edge and spaced 3–5 mm apart across the free edge of the prolapsing leaflet). The knot position is assessed by 3D TEE imaging and a minimum of 3 chordal pairs is recommended to distribute the tension. The rest of the procedure (chordal exteriorization and tensioning) is similar to NC (6, 11) (Figure 4).

In a study by Di Micco et al. published in 2022, many numerical simulations of artificial chordae implantation were done using both devices. They investigated different leaflet insertions and ventricle access sites and they calculated for each simulation the resulting contacting area, tensioning force, and leaflet stress. The chordal implantation was simulated in Barlow's disease (B), forma frusta (FF), and fibroelastic deficiency (FED). They studied different scenarios with different trajectories and different numbers of ePTFE implanted, i.e., from a posterior access site (vertically aligned) corresponding to a reference angle *α* = 0°, to three more anterior access sites, with reference angles equal to 20°, 40°, and 50°. They found that when treating FF prolapses with a NeoChord device, a more anterior LV access site provides larger final displacement values but, on the other side, it has only a small effect on contacting-area, and it is related to tensioning forces and leaflet stress values that can be twice as high compared to those obtained for the posterior LV access site in the FF model. So, with posterior access sites, the risk of leaflet tearing is lower. When they considered the H-MRS, they found out that this device is less affected by the angle for B and FF prolapse. They concluded that (1) the NeoChord device seems to be a preferable solution for reaching the contacting area and maintaining operative leaflet stress if used with a posterior entry site; (2) the H-MRS, although having greater values in terms of tensioning force and leaflet stress, can experience better results if used with an anterior access entry site; (3) for FED prolapse the leaflet sticking point is determinant on the effective restoration of the valve, likely due to the small amount of tissue present in this type of prolapse; (4) there is evidence of contact between anterior leaflet and artificial chordae when the strings are implanted anteriorly (18).

Another important point to consider during this kind of surgery is collaboration between team members. In particular, transesophageal echocardiography represents the “eyes” of the surgeon and a strong interaction with the echo physician (anesthesiologist or cardiologist) is crucial (19, 20).

### 1.5. Learning curve

Like all new techniques, there is a learning curve for NC that requires the acquisition of new technical skills and familiarization with live three-dimensional echocardiographic imaging. In a study by Colli et al., three phases of experience were identified: an initial “learning phase” (first 20 procedures), a second “intermediate phase” and a final “expert phase”. The first phase is characterized by a relatively high actual probability of failure (25%) while the last phase demonstrated a decrease to 5%. However, the cumulative sum control chart (CUSUM) analysis never reached the alarm line beyond which the number of deaths or ineffective procedures would be too high. This condition was also respected at the beginning of the study. This analysis found that a surgeon should complete about 50 procedures in order to reach the “good performance period”. Obviously, this learning curve could be further reduced with a higher amount of proctored procedures and ad-hoc training on ex-vivo programs with simulators (6, 10, 21).

### 1.6. Results and discussion

#### 1.6.1. NeoChord

The TACT trial was published in 2014 and included 30 patients with severe MR due to isolated posterior prolapse that were treated with NeoChord DS1000. During the trial, two procedure refinements were applied that we still use today: the use of multiple neochordae per procedure and the revision of the LV access towards a more posterolateral approach. Almost one-third of patients experienced at least 1 major adverse event (MAE) during the first month. Acute procedural success (APS) was defined as the placement of at least one neochord and reduction of MR to <2+ and this was achieved in 86.7%; at 30 days 58.6% maintained performance success. This was the first trial that demonstrated the feasibility, safety, and efficacy of off-pump transapical implantation of neochords using the NeoChord DS1000. Since this trial, many studies have been conducted over time in order to better understand and improve this new technology and its outcomes. Looking at the acute intraoperative echocardiographic changes, Colli et al. retrospectively analyzed the baseline and early postoperative 3D TEE echocardiography of 66 patients. Immediately after the procedure, they observed a significant reduction (*p* < 0,05) in the systolic anteroposterior diameter, TAPSE, SPAP, indexed left atrial volume, indexed left ventricular end diastolic volume, and left ventricular ejection fraction. At 1 year follow-up, 80.3% of patients presented MR ≤ mild, while 19.7% had MR ≥ moderate. They also observed a reduction of AP diameter, reduction of annulus circumference, reduction of MV area, and reduction of the aorto-mitral angle. These parameters seemed to be protective factors against the recurrence of MR greater than mild. All these changes can be justified by an acute reduction of MR, resulting in an immediate cessation of volume overload in the left atrium and left ventricle that consequently modifies MV geometry over time (22). In 2018, the largest multicenter clinical study after the TACT trial was published. More than 200 patients were enrolled and APS was achieved in 96.7% of patients. In four cases, conversion to conventional surgery for acute recurrence of MR was needed. No intraoperative deaths were observed. Four (1.9%) high-risk patients died within 30 days and these deaths were considered procedure-related (apical rupture, acute respiratory failure, sudden cardiac death, and multiorgan failure). At discharge, of the 206 alive patients, MR was absent/trace in 41.3% of patients, mild in 45.1% of patients, moderate in 12.1% of patients, and severe in 1.5% of patients. At a 30-day follow-up, the incidence of MR was absent/trace, mild, moderate, and severe in 41%, 35.6%, 17.1%, and 6.3%, respectively. During follow up, eight patients underwent MV reintervention for severe MR. At 1 year, MR was absent/trace in 31.4% of patients, mild in 44% of patients, moderate in 16.7% of patients, and severe in 7.9% of patients. At 1 year, the actuarial rate of patients meeting the composite primary endpoint of this study [APS and freedom from death, stroke, structural or functional failure of the MV repair, unplanned intervention related to the procedure, cardiac-related hospitalization, or worsening of the New York Heart Association (NYHA) class] was 84% (DS 2.5%) (9). There are several other studies and reviews that report good clinical and echocardiographic results (3, 23) such as Ahmed et al. with an operative success of 96.8% in 249 patients (24) or in a study by Salihi et al. where APS was achieved in 96.15% of patients; at a 30-month follow-up, freedom from residual severe MR was 78.8% (DS 10.3%) and freedom from reoperation was 87.5% (DS 6.8%) (14).

In the first Greek study by Losoz et al., seven patients with severe mitral regurgitation underwent neochord implantation. At 1 month, five patients showed trivial MR, one patient showed mild MR, and the seventh patient presented severe MR due to severely hypokinetic left ventricular apex. This patient underwent successful PCI to LAD and, 5 months later, their MR grade severity improved to mild-moderate. At a 6-month follow up the same results were reported (25).

A late propensity-matched study comparing transapical beating heart mitral valve repair and conventional surgery (CS) in 372 patients showed interesting data. The study demonstrated the importance of patient selection and its influence on postoperative outcomes in terms of freedom from moderate and severe MR and from reoperation. Freedom from severe MR at follow-up was worse in patients undergoing NC vs. conventional surgery (78.1% and 89.7% respectively, *p* = 0,032) but in patients with type A anatomy, freedom from severe MR between the two groups was similar (79.3% NC vs. 79% CS, *p* = 0,77). Likewise, freedom from reoperation was lower in the NC group (78.9% vs. 92%, *p* = 0.022) but in type A patients, it appeared to be similar between the NC and CS groups (79.7% vs. 85%, *p* = 0.75). The study also could not find any difference in terms of mortality and major postoperative complications between the 2 groups but it showed that all patients had significant improvement in NYHA functional class during follow-up (26).

Regarding long-term results, a recent study published in 2022 showed a 5-year follow-up of 100 neochordae, consecutively implanted. This analysis demonstrated a significant reduction of LV volumes, pulmonary artery pressure, a good midterm safety profile, and satisfactory results in terms of reoperation [16.7% overall and 14.7% with favorable anatomy (FA)] and cumulative incidence of severe residual MR (23.7% overall and 14.7% in patients with FA (13).

In another interesting study, Manzan et al. combined echocardiographic and anatomic variables to predict the outcomes. Their system identified predictors such as LAI, prolapse/flail width, systolic AP and LL mitral annular dimensions, presence of calcification, iLVESV, and SPAP. Then a Cox regression analysis was performed and confirmed the increased hazard of moderate-severe MR related to these variables. This predictive model only supplements clinical decision-making and does not replace good clinical evaluation and/or patient preference. This model is also freely available online at the specific website reported in the study^1^ (27).

#### 1.6.2. Harpoon

The TRACER trial is considered one of the main studies that proved the safety and efficacy of the Harpoon device. It was a prospective non-randomized multicenter trial in which 30 patients with severe MR due to isolated P2 prolapse were enrolled from 2015 to 2017. No perioperative deaths were observed, and the serious adverse events rate was 20% within 30 days. The APS rate was 93% with two intraoperative conversions to traditional surgery. At a 6-month follow-up, 76% of patients presented mild or less MR, 7% with moderate MR, and 7% with severe MR; three patients underwent conventional reoperation for severe MR recurrence. They also observed a positive LV reverse remodeling, a reduction of LV end-diastolic volume, and a reduction of MV anteroposterior diameter at 6 months (19% reduction that was stable at 6-month follow-up) (1, 6). A more recent work by Gammie et al. reported 1-year outcomes after NC with Harpoon in 65 patients. Technical success was achieved in 62 patients (95%). No intraoperative deaths or in-hospital mortality were observed; at discharge the degree of MR was trivial/none in 74%, mild in 21%, moderate in 3%, and severe in 2%. At 30 days, MR severity was none/trivial in 62%, mild in 23%, moderate in 13%, and severe in 2%. One patient required reoperation after 27 days because of severe MR caused by MSSA infective endocarditis. Through 1 year of follow-up, one additional death occurred, one patient experienced a stroke, and no patients experienced endocarditis or renal failure. There was a 13% rate of reoperations in this first year and in all cases the mitral leaflets were intact and the sutures' knot anchors were well incorporated into the tissue. At discharge, 95% of patients had MR less than mild but a progression in the regurgitation to moderate in 13% and to severe in 2% at 30 days was observed. At 1 year, MR was less than mild in 75%, moderate in 23%, and severe in 1% of patients. At 1 year, 98% of patients were in NYHA I or II. As for the NeoChord DS1000, the Harpoon also showed a positive reverse LV and left atrial remodeling was seen. At 1 year, the LV end-diastolic volume was decreased by 22%, LV end-diastolic diameter by 11%, and anterior-to-posterior mitral annular dimension by 11% (11).

### 1.7. Future perspectives

In the last decade, microinvasive mitral valve repair with transapical neochordae implantation has proven to be promising in clinical practice. These procedures allow the surgeon to preserve MV anatomy and to avoid the risks associated with extracorporeal circulation and cardioplegic arrest. They enable to repair the valve on the beating heart during a physiological cardiac cycle and to observe the surgical result in real-time. In the case of MR recurrence, if a conventional surgical operation is needed, there are no issues related to sternal reentry and a surgical repair is still feasible.

Other chordal repair technologies are in their development process, in particular transeptal devices, with the aim to be as minimally invasive as possible and to be used in high-risk patients where other approaches are not feasible.

There are many first-in-human reports available such as the first Mitral Chordal Repair using the transcatheter transeptal NeoChord NeXus System. In a high-risk 55-year-old man, two neochordae were implanted and the mitral coaptation was restored (residual MR was trivial). At a 3-month follow-up, the patient had improved NYHA class and sustained MR reduction and anchor stability. This device has the same advantages as its surgical counterpart and it also has the potential to address both anterior and posterior leaflet pathologies but further cases are required to confirm these initial results (28).

Mitralstitch is a transapical device for beating heart chordal implantation. The pledgeted sutures are implanted directly in the body of the leaflet without a direct suture loop or knots. This device has also a specifically designed retrievable positioning device made of a nitinol frame that provides a precise grasping of the leaflet. The chordae are implanted under TEE imaging and not fluoroscopy. Another feature of Mitralstitch is that this instrument can perform an edge-to-edge procedure through a double leaflet chordal implant and following edge approximation by means of a locker. The results of the first-in-human study published this year by Wang et al. proved the safety and feasibility of this system. Ten patients with severe MR were enrolled and underwent the procedure. MR was reduced from severe to none or trace in five patients, and to mild in five patients at discharge. One patient also received edge-to-edge repair by locking two neochordae. The safety and efficacy endpoint of the study was achieved in all patients at a 1-month follow-up. At 1 year, six patients had mild MR, three had moderate MR, and one had severe MR recurrence and underwent surgical repair (10, 29).

Mistral Chordal Repair is composed of a 12-Fr off-the-shelf catheter and 7.5 Fr delivery system which consists of a 3D nitinol spiral-shaped atraumatic wire inserted from the femoral vein via a transseptal approach to encircle the native mitral chords from both leaflets to bring them together. Chordal approximation is obtained with the rotation of the system. The catheter guide is inserted into the left ventricle via the transfemoral vein and transeptal puncture and then the spiral is released. Under TEE imaging, the surgeon rotates the system in order to catch both leaflet's native chordae and then keeps rotating it in order to move the chordae closer together. When the optimal competency of the valve is achieved, the applicator catheter is retrieved from the ventricle releasing the spiral in the desired final position (6). To the best of our knowledge, no clinical results are available.

ChordArt is a transcatheter mitral repair system and it is made of three components: a distal ventricular/papillary muscle anchor, a proximal nickel-titanium anchor for leaflet securement, and the ePTFE chordae. The procedure is performed through a thoracotomy, but it can be also translated into a percutaneous transfemoral-transeptal delivery system. The leaflet is grasped at the P2 level and is punctured, then the distal anchoring system is delivered to the papillary muscle. In 2021, a study by Weber et al. was published reporting 2-year results. Five patients with symptomatic severe primary degenerative MR were enrolled and treated through a transseptal approach. APS was achieved in all patients and residual MR was trace or absent. MR less than 1+/4 was maintained over 2 years of follow-up. No dehiscence, detachment, or dislocation of the implanted ChordArt devices was observed. The authors also observed a positive remodeling of the left ventricle: the LV end diastolic diameter decreased during the whole follow-up period in comparison to the baseline condition, especially at discharge and 1-month follow-up. The LV end systolic diameter also decreased during the entire follow-up period in comparison to the baseline condition, with an initial mild increase at discharge. Left atrial volume decreased during the follow-up period in comparison to discharge. No major adverse events were reported either during the intervention or during the follow-up period (30).

V-Chordal is a surgical transcatheter technology allowing on-pump chordal implant with off-pump beating heart length adjustment via trans-atrial approach, through a left atrial roof incision. Efficacy and feasibility in animal experiments were demonstrated (31, 32) and there is one study that has been completed on six patients in Europe (including four patients with a complete 1-year follow-up with good results) (6). On clinicaltrials.gov there is one Italian study (Identifier: NCT01415947) that is in phase 2 but no results are yet available.

Pipeline is a transfemoral transseptal chordal repair system for off-pump beating heart chordal replacement. The device has a deflectable guide catheter, a delivery catheter, and four parts that allow chordal implantation: leaflet pledgets with Gore-Tex sutures, a suture lock, a ventricular anchor, and a suture cutter. In 2020, the first-in-human neochord implantation was reported in a 56-year-old patient with symptomatic P2 prolapse and NYHA III. The procedure was successful with echocardiography and fluoroscopy imaging throughout the surgery. The patient had an uneventful immediate postoperative course but before discharge, recurrent asymptomatic MR due to partial anchor displacement was observed and the patient underwent surgical mitral valve replacement (33).

CardioMech is developing a percutaneous solution for artificial chordae implantation. The device is made of a gripper element housing a self-expandable folded anchor made of memory-shape material. When the leaflet is grasped the anchor is unfolded and secured to the leaflet. The system also presents a self-expandable folded papillary anchor made of shape memory metal. The chord length is adjustable under real-time echocardiographic guidance. This technology is in a phase of clinical trials (Identifier: NCT04820764) that is scheduled to be completed in 2026. There is one case report in 2021 of the first-in-human transcatheter transeptal procedure in an elderly patient with severe symptomatic MV regurgitation. The procedure was successful, the regurgitation from severe became mild, and the patient was discharged home the next day (34).

## 2. Conclusions

Transapical off-pump beating heart neochordae implantation is a microinvasive alternative to conventional surgery in patients with severe primary MR. In well-selected patients, this procedure has been shown to be safe and to provide good outcomes for up to 5 years in terms of recurrence of MR and freedom from reoperation. Accurate patient selection based on careful echocardiographic evaluation of anatomic characteristics together with a well-trained surgical team is crucial to obtain good early and long-term clinical and hemodynamic outcomes. At present, there is only one CE-marked device for this operation: NeoChord DS1000. Transapical neochordae implantation has demonstrated that restoring mitral competency in the beating heart by implanting artificial chordae is feasible. As a consequence, several trans-septal devices aimed at implanting neochordae through a completely percutaneous approach are now under development and in the initial investigation phase.

## Footnotes

^1^ https://r-ubesp.dctv.unipd.it/shiny/neochord/

## Supplementary material

SUPPLEMENTARY VIDEO 1

This video shows the main steps of NC implantation.
